# Prevalence and molecular characterization of hepatitis delta virus infection among hepatitis B virus surface antigen positive students and pregnant women in N'djamena, Chad

**DOI:** 10.1016/j.ijregi.2024.100560

**Published:** 2024-12-28

**Authors:** Nalda Debsikréo, Maire Dehainsala, Odan Debsikréo, Nafissatou Leye, Gora LO, Aminata Dia, Makoutchouang Nzonde Biscotine Flore, Ndeye Aminata Diaw, Ndeye Dieynaba Diouf, Isaac Darko Otchere, Rayana Maryse Toyé, Isabelle Chemin, Ali Mahamat Moussa, Ndèye Coumba Toure-Kane, Françoise Lunel-Fabiani

**Affiliations:** 1 Cheikh Anta Diop University, Dakar, Senegal; 2 Institut de Recherche en Santé, de Surveillance Épidémiologique et de Formation, Dakar, Sénégal; 3 University of N'Djamena, N'Djamena, Chad; 4 Centre Hospitalier Universitaire la Référence, N'Djamena, Chad; 5 University of Félix Houphouët Boigny Abidjan, Abidjan, Côte d'ivore; 6 Medical Research Council Unit the Gambia at London School of Hygiene and Tropical Medicine, Fajara, the Gambia; 7 Noguchi Memorial Institute for Medical Research, College of Health Sciences, University of Ghana, Accra, Ghana; 8 Centre de Recherche en Cancérologie de Lyon INSERM U1052, CNRS UMR5286, Université de Lyon, Lyon, France; 9 Centre Hospitalier Universitaire Angers, BAT IBS-4 rue Larrey-49000 ANGERS, Laboratoire HIFIH, UFR Santé département Médecine, SFR 4208-UPRES EA3859, Université d'Angers, Angers Cedex, France

**Keywords:** Hepatitis D virus, Genotype, Prevalence, Students, Pregnant women, Chad and Africa

## Abstract

**Objectives:**

This study sought to determine the prevalence of hepatitis B virus (HBV)-hepatitis D virus (HDV) co-infection and to characterize isolates of both viruses in a Chadian population of HBV surface antigen (HBsAg)-positive pregnant women and students.

**Methods:**

This was a cross-sectional retrospective study using archived samples from pregnant women and students in N'djamena who had been systematically screened for HBsAg between April and August 2021. HBsAg-positive samples were tested for the presence of HDV antibodies (Ab) and were screened for the presence of both HBV and HDV (in anti-HDV Ab-positive samples) viral load estimations. Genome sequencing of the viruses was used for both genotyping and phylogenetic analysis.

**Results:**

A total of 94 participants were included in this study. The mean age was 24 ± 4.89 years (range: 18-42 years). Anti-HDV Ab were found in 9.57% (9/94) of the participants. The prevalence of anti-HDV Ab positivity among students (6.45% [4/62]) was lower than the 15.63% (5/32) observed among pregnant women. HDV-RNA was detected in 7/9 (77.77%) confirmed anti-HDV-positive participants. Most HDV-RN-positive participants had very low HBV DNA viral loads. All HBV sequences belonged to genotype E and all HDV sequences to genotype 1.

**Conclusions:**

Hepatitis D is a potential public health challenge in Chad, which requires active surveillance and public education in the country for proper control. This surveillance should be supported with mass immunization against HBV.

## Highlights

• First genotypic characterization of the hepatitis D virus (HDV) among pregnant women and students in Chad.
• Confirmation of combined presence of hepatitis B virus-E and HDV-1 among co-infected patients.
• High seroprevalence of HDV among pregnant women in Chad.

## Introduction

Hepatitis D virus (HDV) is a hepatotropic virus, a negative-sense circular single-stranded RNA virus of approximately 1.7 kb, and is the only member of the *Deltaviridae* family (genus Deltavirus) [1]. HDV requires hepatitis B virus (HBV) surface proteins (HBsAg) to form infectious viral particles [2]. Without HBV, the HDV is incapable of penetrating hepatocytes and replicating itself [3]. HDV causes acute and chronic liver disease [4] and an increased risk of hepatocellular carcinoma [5].

Delta hepatitis is still a major global health problem affecting 48-60 million individuals worldwide [6]. It is estimated that 7.33% of HBsAg carriers are co-infected or superinfected with HDV in the general population, whereas 9.57% of people with liver disease in West Africa are also infected with HDV [7]. In Central Africa, it is 25.64% of the general population and 37.77% among people with liver disease [7,8].

In addition to Central Africa, regions, including Northern Africa [7], the Amazon basin, Eastern Europe, the Mediterranean countries, the Middle East, and certain regions of Asia, are regions with a high endemicity of HDV [9]. Besides its co-existence with HBV, the HDV genotypes or subtypes may have variable levels of virulence, such as the risk of developing chronic infection. Currently, HDV has eight distinct genotypes, each with two to four subtypes, with a sequence disparity of approximately 35% between genotypes. The predominant genotype in a geographic region varies with genetic diversity between and within genotypes [10]. Genotype 1 is the most widespread and predominates in Europe and North America. Genotype 2 is most widespread in Asia and the Middle East. Compared with genotype 2, genotype 1 has a higher risk of adverse effects and lower remission rates [11]. Genotype 3 is associated with severe liver disease, found in the Amazon basin, and is different from the other genotypes. Genotype 4 is found in Taiwan and China. Genotypes 5, 6, 7, and 8 are predominant in Africa and can also be found in Europe [12].

HDV is mainly transmitted parenterally through blood exposure, unprotected sexual intercourse, or blood products, making mother-to-child transmission very rare [13]. A person may either be simultaneously co-infected with the two hepatitis viruses or be a carrier of HBV prior to exposure to HDV, which is referred to as superinfection [14]. Co-infection or superinfection may lead to fulminant forms or severe chronic course of the disease. It can change the course of asymptomatic or inactive HBV infection into chronic active hepatitis, favoring the development of severe liver lesions and rapid progression to liver cirrhosis and hepatocellular carcinoma [15].

Even though seroprevalence of HDV antibodies (anti-HDV) is 14% among chronic HBsAg carriers in N'Djamena hospitals [16], information regarding the circulating genotypes of HDV is scarcely available in Chad.

Therefore, we sought to determine the prevalence of HDV in a population of HBsAg-positive pregnant women and students using archived samples from previous studies in Chad [17,18] and characterize the detected HBV and HDV.

## Methods

### Study population

This was a retrospective cross-sectional survey of 457 students (both males and females) from the University of N'djamena (public institution) and Emi Koussi University (private institution in N'djamena) Chad from July 3 to July 23, 2021 [17] and 458 pregnant women from eight gynecology and obstetrics departments of hospitals in N'djamena [18]. The inclusion criteria for recruitment are already described in the earlier publications [17,18]. We used only the archived samples from these studies for our study. After taking out seven samples with insufficient sample volume, 94 frozen sera samples positive for HBsAg from 47 males (all students) and 47 females (comprising 15 students and 32 pregnant women) stored at −80 °C in the biobank laboratory of the National Reference Hospital were sent to the “Institut de Recherche en Santé, de Surveillance Epidémiologique et de Formation” (IRESSEF) in Senegal and INSERM U1052 in France for further analysis.

### Serologic assays

Serologic tests were performed at the virology laboratory of the IRESSEF. HBsAg positivity was confirmed using the Abbott Architect i1000SR analyzer (Abbott Diagnostics, Abbott Park, Illinois, USA), and quantification of HDV antibodies (HDV-Ab) was determined using ETI-AB-DELTAK-2 kits (Diasorin, Italy).

#### Nucleic acid extraction

DNA was extracted using the MagMAXTM Viral/Pathogen II Nucleic Acid Kit on the KingFisher™ Flex automated system from Thermo Scientific™, according to the manufacturer's instructions. HBV-DNA and HDV-RNA extractions from samples received at INSERM were performed using the High Pure viral nucleic acid kit (Roche), according to the manufacturer's instructions. The technique requires a volume of 200 µl of serum or plasma, and the nucleic acids were eluted in a final volume of 50 µl.

#### Quantification of hepatitis B virus and hepatitis B virus genotyping

HBV viral load was determined using the GeneProof HBV polymerase chain reaction (PCR) kit with sensitivity up to 13.9 IU/ml and 100% diagnostic sensitivity [19]. Viral DNA of HBV samples had 1200 nt HBV S gene amplified by semi-nested PCR, using the GoTaq® G2 Flexi DNA Polymerase Kit. Two primer pairs, F8 /R5 and F3/R8, were used for PCR 1 and PCR 2 amplification, respectively. The cycling conditions were five initial denaturation at 94 °C, followed by 40 cycles of denaturation at 94 °C for 30 seconds, annealing at 42 °C for 30 seconds, and primer extension at 72 °C for 1 minute before final extension of the amplified DNA at 72 °C for 7 minutes. The PCR products were then visualized on 1.5% agarose gel under ultra violet illumination after staining with ethidium bromide.

#### Quantification of hepatitis D virus and hepatitis D virus genotyping

HDV viral loads (VL) were determined at the Centre de Recherche en Cancérologie de Lyon INSERM U1052 laboratory (France) using ddPCR and reverse transcription-ddPCR (Biorad). One-step quantitative reverse transcription-PCR was performed using the Quantitect Virus kit (Qiagen, Courtaboeuf, France), following the manufacturer's instructions. Co-amplification of CI and HDV RNAs occurred in the same tube. CI detection was performed using the Quasar 670 labeled probe and primers provided in the kit (Simplexa Extraction and Amplification Control Set-RNA; Focus Diagnostics, Cypress, California, and Eurobio, Les Ulis, France) using two direct primers (AgD-F1, AgD-F2) and one reverse primer (AgD-R), following the previously described amplification conditions [20].

#### Sequencing and phylogenetic analyses

To determine HVB and HDV isolates, the HBV *S* gene (1200 nt) and the HDV *R0* region (400 nt) were sequenced by Sanger from purified PCR fragments. The sequenced genetic elements were mapped to respective HBV and HDV reference sequences using the multiple alignment fast Fourier transform (MAFFT) tool. We performed a model test and constructed a phylogenetic tree based on the maximum likelihood method with 1000 bootstrap replicates using the MEGA 7 software.

For alignment and HDV genotyping, prototype HDV-sequences retrieved from the NCBI GenBank were used HDV-1: LS482933, JX888110, JX888098, JX888105, JX888108, U81989, LT604946, KJ744227, MH457142, AM779579, L22066, AM902175, MG711717, MG711801, JX888109, MG711713, MG711667, JN187436; HDV-2: L19598, LT604953; HDV-3: L22063, KC590319; HDV-4: AB118841, AB088679; HDV-5: JX888107, U19598; HDV-6: JX888102, LT604968; HDV-7: AM183333, LT604972, and HDV-8: LT604973, LT604974.

For alignment and HBV-genotyping, HBV-genotype sequences retrieved from the NCBI GenBank were used HBV genotype A: EU694385, KP168423, GQ331046, KM606737, AM184126, FJ692601; genotype B: D23679, JQ801514; genotype C: DQ089781, KC774182; genotype D: AB222711, MF925358; genotype E: KF170742, MT426116, MG621119, MG821154, GQ161817, GQ161772, DQ060824, JQ000008, DQ060822, AJ605008, AM494689; genotype F: HM585194, DQ899143; genotype G: AB056513; genotype H: FJ356715, and genotype I: AB562463; FJ023669.

### Statistical analysis

The obtained data were entered into Microsoft Excel and imported into STATA version 12.1 for data analysis. Quantitative variables were expressed as mean ± SD. Categoric variables were expressed as numbers and percentages and compared using chi-square or Fisher's exact test when appropriate, whereas the independent *t*-test was used to compare the means of continuous variables. When normal distribution or equal variance could not be assumed, the Mann-Whitney U test was used. With a 95% confidence interval (CI), *P*-values <0.05 were considered statistically significant.

## Results

### Characteristics of the study population

As shown below (Table 1), a total of 94 samples from 62 students and 32 pregnant women described elsewhere [17,18] (mean age 24.39 ± 4.89 years) were included in this study. In addition to the 32 pregnant women, there were 15 females among the students whose samples were used for this study, given a 1:1 ratio. Whereas all the pregnant (100%) women were married, only 16 (25.8%) of the students were married. Seventy-one (75.53%) of the participants had had unprotected sex, 25 (26.60%) had HBsAg-positive mothers, and other potential risk factors for HBV/HDV infection, including family history of HBV, blood transfusions, transcutaneous medical examinations, and injecting drugs.

**Table 1:** Participant's sociodemographic characteristics and history.

Variable	Pregnant women n = 32	Student n = 62	Total cohort n = 94
Age	Mean = 25	Mean = 23	Mean = 24.39 (± 4.89)
18-24	15 (46.88)	42/62 (67.74)	57 (60.64)
25-34	11 (34.38)	20/62 (32.26)	31 (32.98)
≥35	6 (18.75)	0/62 (0.00)	6 (6.38)
Gender			
Male	0 (00.00%)	47 (75.81)	47 (50.00)
Female	32/32 (100.00)	15 (24.19)	47 (50.00)
Marital status			
Single	0/32 (0.00)	46/62 (74.19)	46 (48.94)
Married	32/32 (100.00)	16/62 (25.81)	48 (51.06)
Potential risk factors			
Blood transfusions	12/32 (12.50)	10/62 (16.13)	14/94 (14.89)
Hospital admission	8/32 (25.00)	13/62 (20.97)	21/62 (22.34)
Mother hepatitis B surface antigen positive	4/32 (12.50)	21/62 (33.87°	25/94 (26.60)
Family history hepatitis B virus	2/32 (6.25)	13/62 (20.97)	15/94 (15.96)
Transcutaneous medical examinations	0/32 (00.00)	16/62 (25.81)	16/94 (17.02)
Unprotected sex	31/32 (96.88)	20/62 (64.52)	71/94 (75.53)
Injection drugs	0/32 (00.00)	1/62 (1.61)	1/94 (1.06)
Jail	1/32 (3.13)	5/62 (8.06)	6/94 (6.38)

### Anti-hepatitis D virus antibodies seroprevalence and associated risk factors

The HDV prevalence (anti-HDV antibodies) of the total cohort was 9.57% (9/64), whereas that of the students and the pregnant women was 6.45% (4/62) and 15.63% (5/32), respectively (Table 2).

**Table 2:** Prevalence of hepatitis D virus among participants.

Variable	Categories	Anti-hepatitis D virus antibodies		Crude odds ratio	*P* (95% confidence interval)
		Negative	Positive		
Cohort	Pregnant women	27 (84.38)	5 (15.63)	0.37	0.265 (0.09-1.49)
	Student	58 (93.55)	4 (6.45)		
Age				1.31	0.607 (0.45-3.77)
	18-24	52 (91.23)	5 (8.77)		
	25-34	28 (90.32)	3 (9.68)		
	≥35	5 (83.33)	1 (16.67)		
Gender				0.78	1.000 (0.19-3.11)
	Female	42 (89.36)	5 (10.64)		
	Male	43 (91.49)	4 (8.51)		
Marital situation				1.22	1.000 (0.30-4.86)
	Single	42 (91.30)	4 (8.70)		
	Married	43 (89.58)	5 (10.42)		
Blood transfusions				1.73	0.618 (0.32-9.38)
	No	73 (91.25)	7 (8.75)		
	Yes	12 (85.71)	2 (9.57)		
Hospital admission				0.40	0.678 (0.04-3.44)
	No	65 (89.04)	8 (10.96)		
	Yes	20 (95.24)	1 (4.76)		
Hepatitis B surface antigen positive mother				1.25	0.656 (0.47-3.33)
	No	55 (91.67)	5 (8.33)		
	Yes	22 (88.00)	3 (12.00)		
Family history hepatitis B virus				0.37	0.191 (0.11-1.28)
	No	46 (85.19)	8 (14.81)		
	Yes	15 (100.00)	0 (0.00)		
Transcutaneous medical examinations				NA	NA
	No	69 (88.46)	9(11.54)		
	Yes	16 (100.00)	0 (0.00)		
Unprotected sex				2.79	0.445 (0.33-23.62)
	No	22 (95.65)	1 (4.35)		
	Yes	63 (88.73)	8 (11.27)		
Injection drugs				NA	NA
	No	85 (91.40)	8 (8.60)		
	Yes	0 (0.00)	1 (100.00)		
Jail				NA	NA
	No	79 (89.77)	9 (10.23)		
	Yes	6 (100.00)	0 (0.00)		

### Detection and quantification of hepatitis B virus DNA and hepatitis D virus RNA

**Figure 1:**
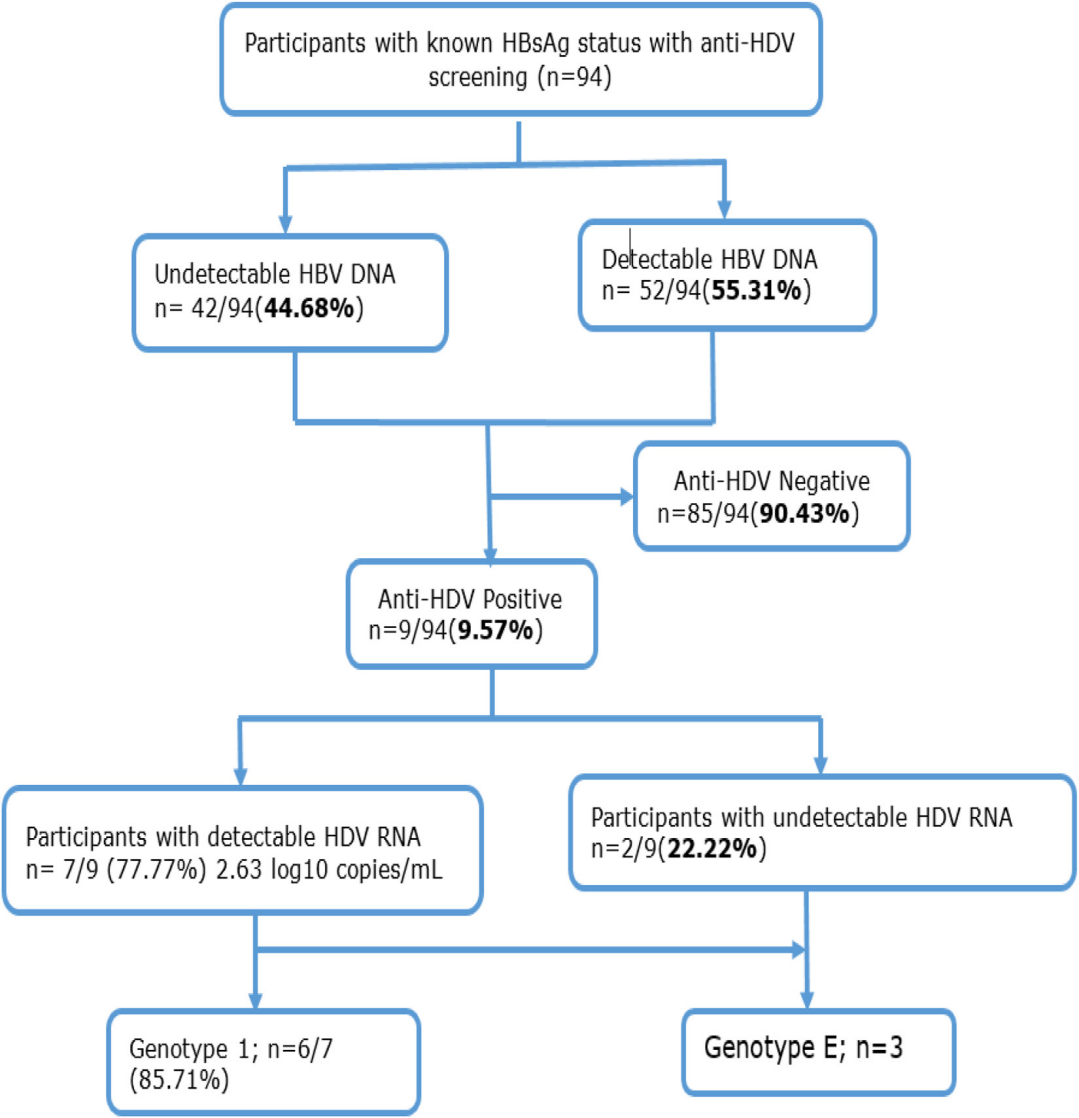
Flow chart of study participants.

HBsAg, hepatitis B surface antigen; HBV, hepatitis B virus; HDV, hepatitis D virus.

HBV DNA was detected in 52 of the 94 HBsAg-positive cases (55.31%) and HDV RNA in 7/9 (77.77%) of the anti-HDV-Ab-positive participants. The median HDV viral load (VL) was 2.63 log10 copies/ml (1.18-3.22), with only one sample having a VL greater than 3 log10 copies/ml with three samples having a VL greater than 3 log10 copies/ml (Figure 1).

### Hepatitis B virus and hepatitis D virus genotypes

**Figure 2:**
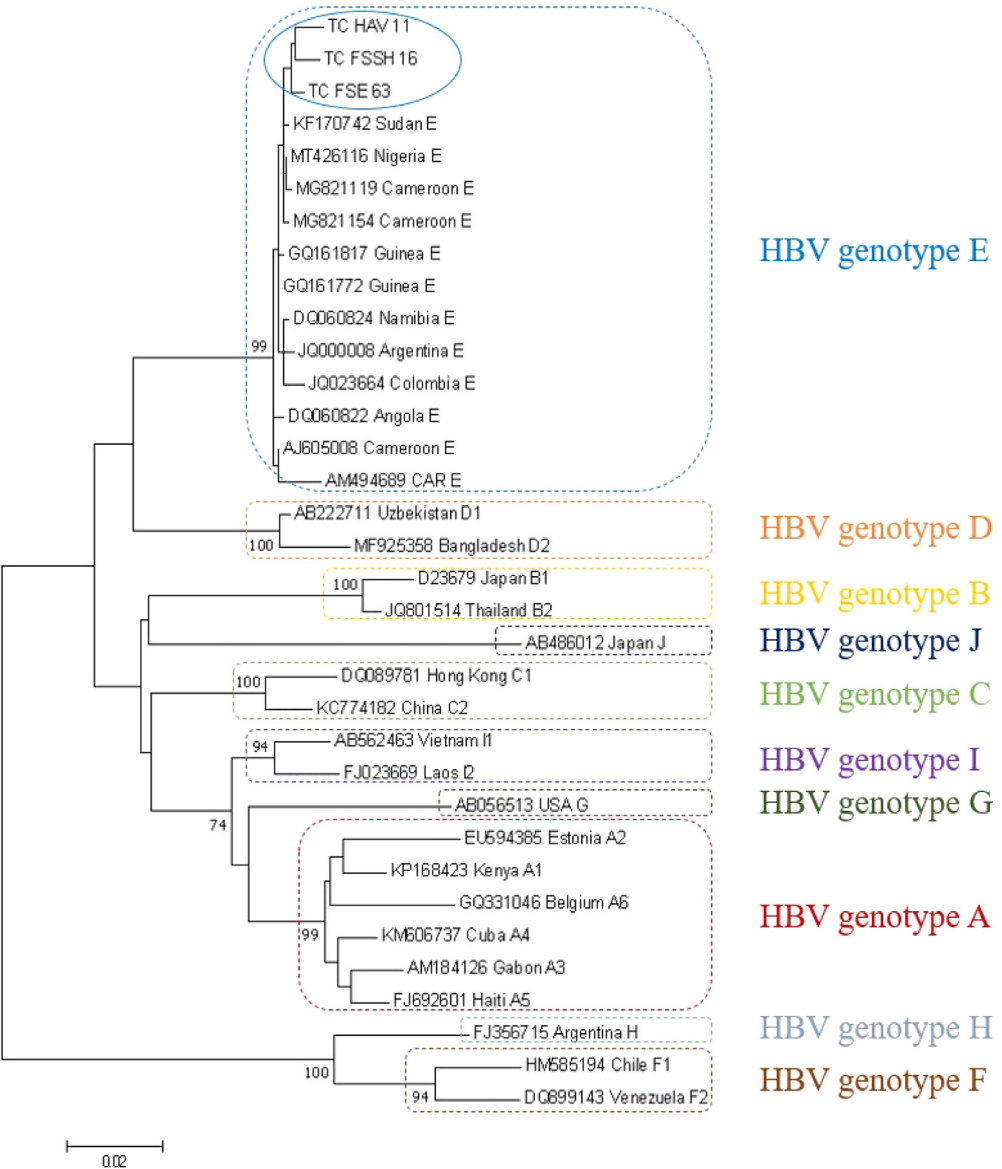
Maximum likelihood phylogenetic tree of Chadian HBV.

HBV genotyping was successfully performed for three samples, including those from one pregnant woman and two male students. The model test showed that the best substitution model with the lowest Bayesian information criterion (BIC) score was the Kimura-2 parameter using a discrete Gamma distribution with five rate categories (K2+G). The phylogenetic tree comprised our three sequences and 31 HBV reference sequences from all genotypes (34 sequences in total). Figure 2 shows that all HBV sequences from Chad belong to genotype E. Indeed, our sequences clustered close to HBV genotype E reference sequences with a very high bootstrap value (99%) and closer to genotype E sequences from neighboring countries, including Sudan, Nigeria, and Cameroon.

Isolates (981 nucleotides) and HBV genotypes A to J reference sequences retrieved from the GenBank database. The evolutionary history was inferred using the maximum likelihood method based on the Kimura 2-parameter (K2) model. Samples sequenced in this study are identified by the prefix TC. Reference sequences are identified by their accession number, country of origin, and (sub) genotype. The numbers at each node correspond to the bootstrap values (>70%) obtained with 1000 replicates. HBV, hepatitis B virus.

**Figure 3:**
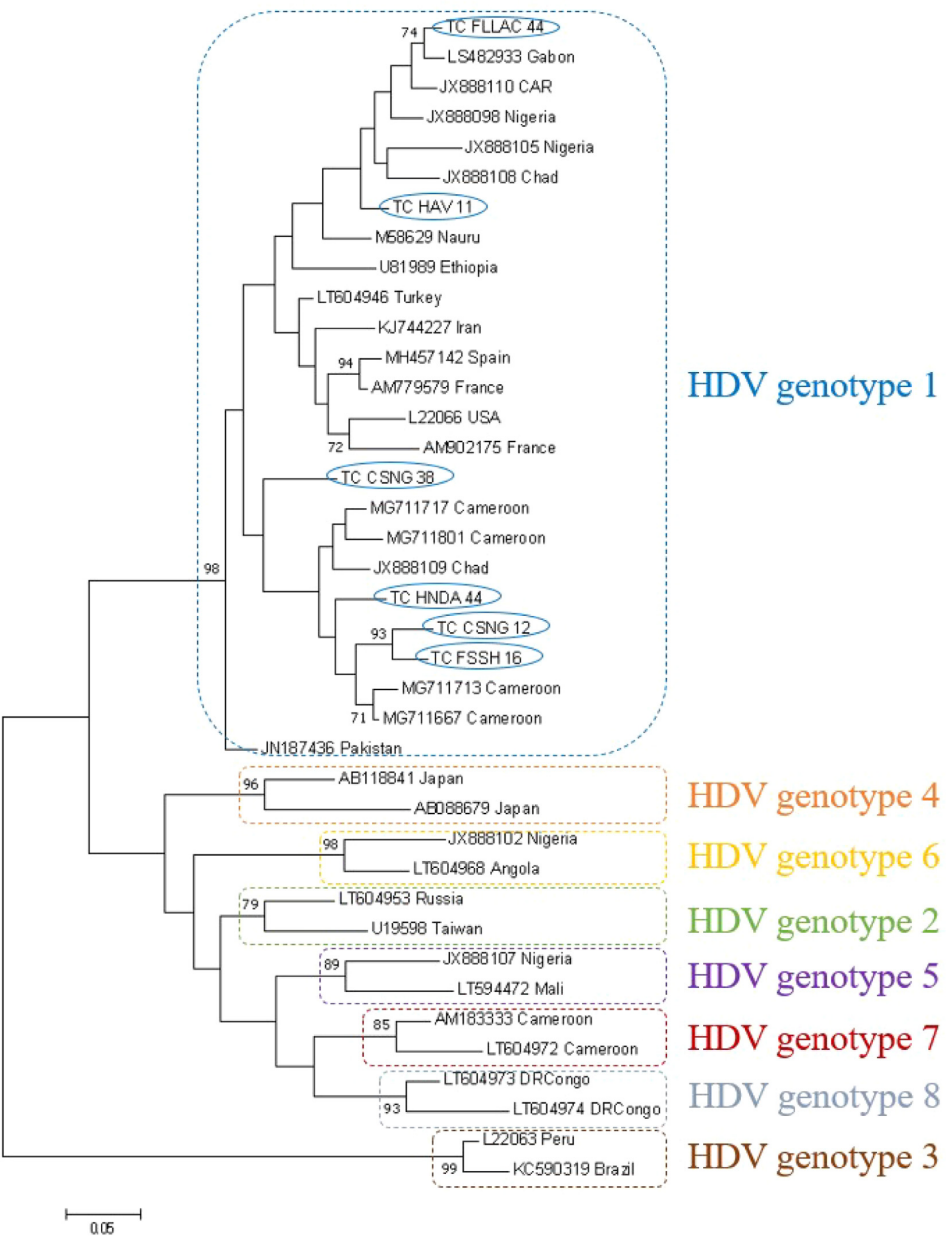
Maximum likelihood phylogenetic tree of Chadian HDV.

HDV genotyping was successful in six samples, including three pregnant women, one female student, and two male students. The model test showed that the best substitution model with the lowest BIC score was the Tamura-3 parameter using a discrete Gamma distribution with five rate categories (T92+G). The phylogenetic tree comprised our six sequences and 33 HDV reference sequences from all genotypes (39 sequences in total). Figure 3 shows that all HDV sequences from Chad belong to genotype 1. Indeed, our sequences clustered close to HDV genotype 1 reference sequences with a very high bootstrap value (98%) and closer to genotype 1 sequences from neighboring countries, including Cameroon, Nigeria, Central African Republic (CAR), and Gabon.

Isolates (367 nucleotides) and HDV genotypes 1 to 8 reference sequences retrieved from the GenBank database. The evolutionary history was inferred using the maximum likelihood method based on the Tamura 3-parameter (T92) model. Samples sequenced in this study are identified by the prefix TC. Reference sequences are identified by their accession number and country of origin. The numbers at each node correspond to the bootstrap values (>70%) obtained with 1000 replicates. HDV, hepatitis D virus.

Two patients (an 18-year-old pregnant woman and a 20-year-old male student) were both co-infected with the HBV-E and HDV-1 combination.

The HBV and HDV nucleotide sequences generated from this study were deposited in the GenBank database accession numbers PQ252377- PQ252379 and PQ112532- PQ112537, respectively.

## Discussion

Co-infection of HBV and HDV poses a significant public health threat, and there is limited data on the prevalence of HDV in Chad. Therefore, this study sought to estimate the prevalence of HDV infection among pregnant women and students in Chad. Notwithstanding the small sample size used in this study, these populations are important because it is possible to limit the spread of infection through protective measures such as vaccination and transmission of mother to child. The seroprevalence of anti-HDV Ab was searched in a cohort of 94 individuals who tested positive for HBsAg, and molecular characterization of HBV and HDV was performed in the HBsAg-positive and HDV-positive samples.

The overall prevalence of anti-HDV Ab in the HBsAg-positive cohort was 9.57%, which was low compared with the study carried out in N'Djamena hospitals, which reported an overall prevalence of 14% [16] but consistent with other reports from other African countries with a prevalence between 4% and 15% [7,21]. Our results show that Chad is an HDV-endemic country, which aligns with the report that most African countries have a high HDV endemicity [7]. The prevalence of anti-HDV Ab among pregnant women was higher (15.63% [5/32]) compared with students (6.45% [4/62]), although the difference was not significant (*P* = 0.265, 95% confidence interval 0.09-1.49). Nevertheless, this finding highlights that HDV infection in pregnant women might be a significant health problem, consistent with earlier reports [22,23].

The prevalence of HDV antibodies rate of 15.63% among pregnant women in this study is consistent with the results of a study conducted in Gabon, which reported a prevalence of 15.6% in pregnant women [24] and another in Mauritania with 14.7% prevalence [25]. However, it is lower than the 20.63% reported in Pakistan [26] but higher than the 7.3% reported in Cameroon [27]. Similarly, the observed 6.45% HDV prevalence among students in this study compares to the 5.4% reported among Central African students [28]. It can also be said that students are younger and less often exposed to nosocomial contamination, which is often the mode of contamination for HDV [25].

HDV RNA load was detected in 77.77% of anti-HDV-positive participants, of whom the majority had very low HBV DNA load. HBV DNA was detected in 55.32% of the HBsAg-positive individuals, confirming active HBV infection. Interestingly, only 87.5% of anti-HDV-positive participants also tested positive for HBV DNA. Moreover, HDV RNA was detected in 77.77% of anti-HDV-positive participant samples. These results are consistent with the available literature showing that HDV replication is found in approximately 50% of anti-HDV-positive participants and also agree with the assertion that HDV infection suppresses HBV replication [2]. These findings highlight the importance of testing for both HBV DNA and HDV RNA to accurately assess the status of HBV and HDV infection.

HDV can be transmitted by drug injection practices, including poorly sterilized needles and medical instruments, re-use of disposable needles and syringes, history of transfusion, and unprotected sexual intercourse [15]. Although several of such risk factors for HDV infection were explored in this study, we did not find statistically significant associations between these factors and HDV seropositivity among our small sample size. This discovery may explain further why pregnant women were more likely to be HDV-positive compared with the students, with the hypothesis that the pregnant women were more likely to engage in unprotected sex compared with the students who, even if sexually active, do engage in protected sex. A second hypothesis can also be that students are younger and less often exposed to nosocomial contamination, which is often the mode of contamination for HDV. However, these hypotheses call for further research with a larger cohort for better exploration of the mentioned risks and other potential risk factors associated with HDV infection in Chad.

All the HBV sequences generated in our study belonged to genotype E, which is found almost exclusively in Sub-Saharan Africa. HBV from our study clustered with HBV from neighboring countries (Sudan, Nigeria, and Cameroon), indicating the circulation of region-specific HBV genotypes. All the HDV sequences generated in our study belonged to the globally ubiquitous genotype 1. Interestingly, our sequences clustered into two distinct groups within HDV-1, comprising sequences isolated from the same country (Chad) or neighboring countries (Cameroon, Nigeria, CAR, and Gabon).

### Limitations and future directions

Our study was limited by the small sample size, which may affect the generalizability of the results. In addition, the retrospective nature of our study made it impossible to, unfortunately, get clinical data. Further research, including longitudinal studies with larger cohorts, is needed to better understand the epidemiology of HDV infection in Chad and to develop effective prevention and control strategies.

## Conclusion

Despite the small sample size, the results of this study confirm the high prevalence of HDV, particularly among pregnant Chadian women (>10%). In addition, HBV and HDV genotyping showed that HBV-E and HDV-1 circulate in this region as expected. Finally, a combination of genotypes of both viruses was found in young adults. This study reported a prevalence of HDV of 15.63% in pregnant women and 6.45% in students. HDV viral RNA was detectable in 77.77% of the participants. In Chad, there is certainly a need to test HBsAg-positive pregnant women for total antibodies against HDV, as positive ones might present more severe liver disease.

## Declarations of competing interest

The authors have no competing interests to declare.

## References

[1] Botelho-Souza LF, Vasconcelos MPA, dos Santos A de O, Salcedo JMV, Vieira DS (2017). Hepatitis delta: virological and clinical aspects. Virol J.

[2] Shirvani-Dastgerdi E, Tacke F. (2015). Molecular interactions between hepatitis B virus and delta virus. World J Virol.

[3] Caviglia GP, Ciancio A, Rizzetto M. (2022). A review of HDV infection. Viruses.

[4] Wedemeyer H, Manns MP. (2010). Epidemiology, pathogenesis and management of hepatitis D: update and challenges ahead. Nat Rev Gastroenterol Hepatol.

[5] Kamal H, Fornes R, Simin J, Stål P, Duberg AS, Brusselaers N (2021). Risk of hepatocellular carcinoma in hepatitis B and D virus co-infected patients: A systematic review and meta-analysis of longitudinal studies. J Viral Hepat.

[6] Miao Z, Zhang S, Ou X, Li S, Ma Z, Wang W (2020). Estimating the global prevalence, disease progression, and clinical outcome of hepatitis delta virus infection. J Infect Dis.

[7] Stockdale AJ, Chaponda M, Beloukas A, Phillips RO, Matthews PC, Papadimitropoulos A (2017). Prevalence of hepatitis D virus infection in sub-Saharan Africa: a systematic review and meta-analysis. Lancet Glob Health.

[8] Lunel-Fabiani F, Mansour W, Amar AO, Aye M, Le Gal F, Malick FZF (2013). Impact of hepatitis B and delta virus co-infection on liver disease in Mauritania: A cross sectional study. J Infect.

[9] Hayashi T, Takeshita Y, Hutin YJF, Harmanci H, Easterbrook P, Hess S (2021). The global hepatitis delta virus (HDV) epidemic: what gaps to address in order to mount a public health response?. Arch Public Health.

[10] Le Gal F, Brichler S, Drugan T, Alloui C, Roulot D, Pawlotsky JM (2017). Genetic diversity and worldwide distribution of the Deltavirus genus: a study of 2,152 clinical strains. Hepatology.

[11] Su CW, Huang YH, Huo TI, Shih HH, Sheen IJ, Chen SW (2006). Genotypes and viremia of hepatitis B and D viruses are associated with outcomes of chronic hepatitis D patients. Gastroenterology.

[12] Sagnelli C, Pisaturo M, Curatolo C, Codella AV, Coppola N, Sagnelli E. (2021). Hepatitis B virus/hepatitis D virus epidemiology: changes over time and possible future influence of the SARS-CoV-2 pandemic. World J Gastroenterol.

[13] World Health Organization (2024). https://www.who.int/news-room/fact-sheets/detail/hepatitis-d.

[14] Khabir M, Aliche AZ, Sureau C, Blanchet M, Labonté P. (2020). Hepatitis delta virus alters the autophagy process to promote its genome replication. J Virol.

[15] Farci P, Niro GA, Zamboni F, Diaz G. (2021). Hepatitis D virus and hepatocellular carcinoma. Viruses.

[16] Moussa AM, Saleh TM, Habkreo M, Nadlaou B, Ngare AA, Stanislas DA (2022). Prevalence and predictors of viral hepatitis D co-infection in chronic HBsAg carriers. Open J Gastroenterol.

[17] Debsikréo N, Mankréo BL, Moukénet A, Ouangkake M, Mara N, Moussa AM (2024). Prevalence of hepatitis B virus infection and its associated factors among students in N'Djamena, Chad. PLoS One.

[18] Debsikréo N, Mankréo BL, Moukenet A, Ndiaye AJS, Diouf NL, Lo G (2023). Prevalence of hepatitis B and associated factors among pregnant women in N'Djamena, Chad. Fortune J Health Sci.

[19] s.r.o VA. GeneProof hepatitis B virus (HBV) PCR testing, http://www.geneproof.com/geneproof-hepatitis-b-virus-hbv-pcr-kit/p1093; 2024 [accessed 16 May 2024].

[20] Scholtes C, Icard V, Amiri M, Chevallier-Queyron P, Trabaud MA, Ramière C (2012). Standardized one-step real-time reverse transcription-PCR assay for universal detection and quantification of hepatitis delta virus from clinical samples in the presence of a heterologous internal-control RNA. J Clin Microbiol.

[21] Stockdale AJ, Kreuels B, Henrion MYR, Giorgi E, Kyomuhangi I, de Martel C (2020). The global prevalence of hepatitis D virus infection: systematic review and meta-analysis. J Hepatol.

[22] Eyi ASA, Komba OM, Chambellant C, Boussoukou IPM, Moukambi L, Moukambi KMWA (2024). Triple burden of hepatitis B, hepatitis Delta viruses, and Plasmodium falciparum to pregnant women. IJID Reg.

[23] Ndzie Ondigui JL, Mafopa Goumkwa N, Lobe C, Wandji B, Awoumou P, Voussou Djivida P (2024). Prevalence and risk factors of transmission of hepatitis delta virus in pregnant women in the Center Region of Cameroon. PLoS One.

[24] Makuwa M, Caron M, Souquière S, Malonga-Mouelet G, Mahé A, Kazanji M. (2008). Prevalence and genetic diversity of hepatitis B and delta viruses in pregnant women in Gabon: molecular evidence that hepatitis delta virus Clade 8 originates from and is endemic in Central Africa. J Clin Microbiol.

[25] Mansour W, Malick FZF, Sidiya A, Ishagh E, Chekaraou MA, Veillon P (2012). Prevalence, risk factors, and molecular epidemiology of hepatitis B and hepatitis delta virus in pregnant women and in patients in Mauritania. J Med Virol.

[26] Aftab M, Naz S, Aftab B, Ali A, Rafique S, Fatima Z (2019). Characterization of hepatitis delta virus among pregnant women of Pakistan. Viral Immunol.

[27] Ducancelle A, Abgueguen P, Birguel J, Mansour W, Pivert A, Le Guillou-Guillemette H (2013). High endemicity and low molecular diversity of hepatitis B virus infections in pregnant women in a rural district of North Cameroon. PLoS One.

[28] Komas NP, Ghosh S, Abdou-Chekaraou M, Pradat P, Al Hawajri N, Manirakiza A (2018). Hepatitis B and hepatitis D virus infections in the Central African Republic, twenty-five years after a fulminant hepatitis outbreak, indicate continuing spread in asymptomatic young adults. PLOS Negl Trop Dis.

